# Remote Ischemic Preconditioning for the Prevention of Contrast-Induced Acute Kidney Injury in Diabetics Receiving Elective Percutaneous Coronary Intervention

**DOI:** 10.1371/journal.pone.0164256

**Published:** 2016-10-10

**Authors:** Gillian Balbir Singh, Soe Hee Ann, Jongha Park, Hyun Chul Chung, Jong Soo Lee, Eun-Sook Kim, Jung Il Choi, Jiho Lee, Shin-Jae Kim, Eun-Seok Shin

**Affiliations:** 1 Department of Cardiology, Ulsan University Hospital, University of Ulsan College of Medicine, Ulsan, South Korea; 2 Division of Nephrology, Ulsan University Hospital, University of Ulsan College of Medicine, Ulsan, South Korea; 3 Division of Endocrinology, Ulsan University Hospital, University of Ulsan College of Medicine, Ulsan, South Korea; 4 Department of Occupational & Environmental Medicine, Ulsan University Hospital, University of Ulsan College of Medicine, Ulsan, South Korea; Kurume University School of Medicine, JAPAN

## Abstract

**Objective:**

Remote ischemic preconditioning (RIPC) induces transient episodes of ischemia by the occlusion of blood flow in non-target tissue, before a subsequent ischemia-reperfusion injury. When RIPC is applied before percutaneous coronary intervention (PCI), the kidneys may be protected against ischemia-reperfusion injury and subsequently contrast-induced acute kidney injury (CI-AKI). The aim of this study was to evaluate the efficacy of RIPC for the prevention of CI-AKI in patients with diabetes with pre-existing chronic kidney disease (CKD) undergoing elective PCI.

**Methods:**

This randomized, double-blind, sham-controlled study enrolled patients with diabetes scheduled for elective PCI with eGFR ≤60 ml/min/1.73 m^2^ or urinary albumin creatinine ratio of >300 mg/g to receive either RIPC or the sham ischemic preconditioning.

**Results:**

One hundred and two patients (68.9 ± 8.2 years old, 47.1% men) were included. Baseline eGFR, creatinine and serum NGAL was similar between RIPC and control groups (48.5 ± 12 ml/min vs. 46.6 ± 10 ml/min, *p* = 0.391; 1.42 ± 0.58 mg/dl vs. 1.41 ± 0.34 mg/dl, *p* = 0.924; and 136.0 ± 45.0 ng/ml vs. 137.6 ± 43.3 ng/ml, *p* = 0.961, respectively). CI-AKI occurred in 13.7% (14/102) of the total subjects, with both RIPC and control groups having an equal incidence of 13.7% (7/51). No significant differences were seen in creatinine, NGAL, cardiac enzymes (troponin T, CKMB) and hs-CRP between the groups post-procedure.

**Conclusions:**

In this study, RIPC applied prior to elective PCI was not effective in preventing CI-AKI in patients with diabetes with pre-existing CKD.

**Trial Registration:**

ClinicalTrials.gov NCT02329444

## Introduction

Contrast-induced acute kidney injury (CI-AKI) is a significant iatrogenic complication of contrast media, which is associated with prolonged hospitalization, cardiovascular events, persistent kidney damage and increased mortality [1, 2] and accounts for up to 15% of all cases of hospital-acquired AKI [3, 4]. Diabetes with pre-existing renal disease can further increase the risk of CI-AKI [2, 4, 5]. The usual clinical course of CI-AKI is an increase in serum creatinine levels within 48 hours of contrast exposure, peaking at 3 to 5 days, before gradually returning to baseline within 1 to 3 weeks.

Remote ischemic preconditioning (RIPC) is a non-pharmacological strategy inducing transient episodes of ischemia by the occlusion of blood flow in non-target tissue such as a limb before a subsequent ischemia-reperfusion injury occurs in a more distant organ [6]. These brief, repeated ischemic episodes can confer protection at more remote sites such as the heart, brain, lung, kidney, intestine or skeletal muscle via an adaptational response that protects against the ischemia and reperfusion insult [6–8]. The protective mechanism of RIPC arises from the complex interactions involving respective signal transduction, anti-inflammatory, neuronal and humoral pathways differing in response to various ischemic stimuli [6, 8]. The kidney is especially sensitive to ischemic injury. Therefore, when RIPC is applied before percutaneous coronary intervention (PCI), the kidneys may be protected against ischemia-reperfusion injury and subsequently CI-AKI [9]. The objective of this study is to evaluate the efficacy of RIPC in preventing CI-AKI in patients with diabetes with pre-existing chronic kidney disease (CKD) undergoing PCI.

## Materials and Methods

### Subjects

This prospective, randomized, double-blind, sham-controlled study was performed at Ulsan University Hospital, South Korea between March 2012 and January 2015. Patients with type 2 diabetes with pre-existing CKD [estimated glomerular filtration rate (eGFR) <60 ml/min/1.73m^2^ or random urine albumin-to-creatinine ratio >300 mg/g] presenting with chest pain and undergoing elective PCI were included. eGFR was calculated based on the creatinine level using the Modification of Diet in Renal Disease equation [10]. Patients with ST-elevation myocardial ischemia (STEMI), decompensated heart failure in the preceding 6 months, patients with end stage renal disease on maintenance dialysis, cerebrovascular disease, chronic liver disease, chronic obstructive pulmonary disease, gastrointestinal bleeding, acute or chronic infection or malignancy were excluded. This study complies with the Declaration of Helsinki and was approved by the Institutional Review Board (IRB) ethics committee of Ulsan University Hospital on 16^th^ January 2012. Patient recruitment began on the 23^rd^ March 2012 until 27^th^ January 2015. Follow up was complete 72 hours after enrolled patients received their percutaneous coronary intervention. All participants provided written, informed consent. The study protocol is described in S1 and S2 Files. The CONSORT checklist for randomised trial reporting is shown in S3 File. The Clinicaltrials.gov registration number is NCT02329444. There was an unintentional delay in registering the clinical trial until after enrolment of participants started and there was no exposure of preliminary results or bias induced from this delay. The authors confirm that there are no further ongoing study trials for this intervention.

### Randomization

**Fig 1:**
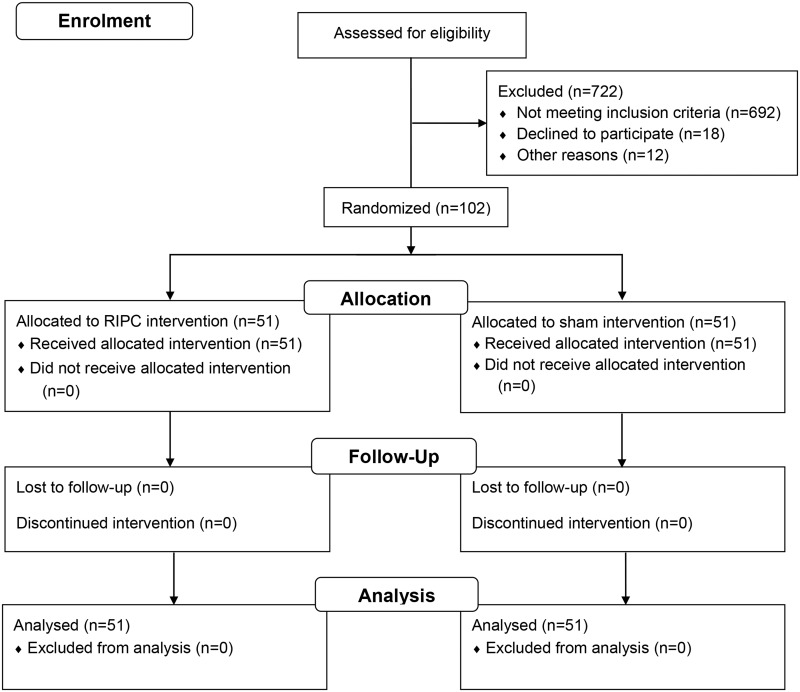
The CONSORT flow diagram. This shows the flow of participants through each stage in this randomized trial.

A computer-generated block randomization stratified for age and sex was used to randomly assign consecutive patients in a 1:1 ratio to RIPC or control group. All patients, study investigators and interventionalists were blinded to the allocation. Only an independent nurse performing the RIPC and sham preconditioning knew the group allocations. The CONSORT flow diagram depicting the flow of participants through each stage of this randomized trial is shown in Fig 1.

### Procedures

**Fig 2:**
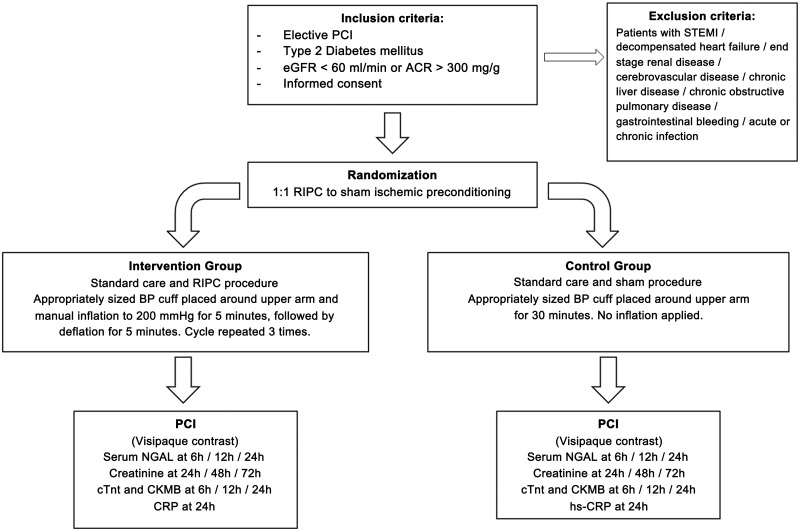
Study procedures diagram. Patients meeting the inclusion criteria were randomized to receive remote ischemic preconditioning or sham ischemic preconditioning 30 minutes before the start of percutaneous coronary intervention. *Abbreviations*: RIPC, remote ischemic preconditioning; PCI, Percutaneous coronary intervention; NGAL, neutrophil gelatinase-associated lipocalin; cTnT, cardiac troponin T; hs-CRP, high sensitivity C-reactive protein; CKMB, creatinine kinase MB.

The study procedures diagram is shown in Fig 2. Patients received pre-hydration with intravenous 0.9% NaCl infusion at 60 ml/hour 6 hours before and after PCI procedure. *N*-acetylcysteine was not administered to any patient. Nephrotoxic agents, anti-inflammatories, metformin, diuretics and renin-angiotensin blockers were discontinued the day before the procedure. RIPC or sham preconditioning was performed 30 minutes before the PCI. All patients had an appropriately sized sphygmomanometer cuff placed around their right upper arm (where contraindicated, the left arm). RIPC was performed by manual inflation of the cuff to 200 mmHg for 5 minutes, followed by deflation of 5 minutes to allow reperfusion and this cycle was performed 3 times. The correct cuff inflation was verified by the disappearance of a pulsatile signal on pulse oximeter placed on the ipsilateral index finger [11]. The sham group had the application of the cuff for 30 minutes with no inflation applied. All patients were treated with acetylsalicylic acid 200mg and clopidogrel 300 to 600mg loading dose before the procedure, and 100U/Kg of unfractionated heparin was injected intravenously to maintain an activated clotting time ≥250s during the procedure. Iodixanol (Visipaque 320 mgI/ml solution), an iso-osmolar contrast media injection was used for all patients. Patients received coronary angiography as per standard procedures at our hospital and stenting of diseased vessels were at the discretion of the operator.

### Outcomes

Serum creatinine level was measured at 24, 48 and 72 hours post PCI. Serum neutrophil gelatinase-associated lipocalin (NGAL), cardiac troponin T (cTnT) and CK-MB was measured at baseline, 6, 12 and 24 hours, and high sensitivity C-reactive protein (hs-CRP) at baseline and 24 hours post procedure. NGAL was measured using the NGAL ELISA Kit (BioPorto Diagnostics A/S, Denmark). The primary outcome was CI-AKI, defined as a creatinine rise of ≥0.5 mg/dl from baseline and/or an increase in creatinine of ≥25% from baseline within 48 hours after contrast exposure [12–14]. Secondary outcomes were the serial changes in creatinine, NGAL level, cTnT, CKMB and hs-CRP over time from baseline.

### Statistical analysis

Categorical variables were described as frequencies and proportions and were compared with the Chi-square tests or Fisher’s exact test. Continuous variables were presented as means ± standard deviation. The differences in means of continuous measurements were examined using the independent samples *t*-test and non-parametric Mann-Whitney U test as appropriate. All *p*-values were 2-sided analysis with statistical significance level of 0.05. As previous literature has estimated the incidence of CI-AKI between 20–30%, we based our study sample on this estimate, keeping in mind that the patient characteristics and study population were different. Using the risk reduction findings of 30% on the success of RIPC to reduce the incidence of AKI in the intervention arm compared to the control group from a previous study [9], and a study power of 0.80 and 2-sided significance level (α) level of 0.05, it was calculated that at least 50 patients were needed in each arm of the study. Statistical analyses were done using SPSS (version 21.0; SPSS Inc, Chicago, Illinois).

## Results

102 patients (mean age 68.9 ± 8.2 years old, 47.1% men) were included in this study. The baseline clinical characteristics and laboratory measurements are shown in Tables 1 and 2 respectively. The volume of contrast media was similar between RIPC and control groups (197.5 ± 114.3 mls vs. 196.4 ± 118.8 mls, *p* = 0.960). Over a third of patients, 31.4% in the RIPC group and 35.3% in the control group received more than 200 mls of contrast. The Mehran’s score (probability of developing CI-AKI) was high or very high in 52 patients (51.0%) with a median score of 11, predicting a 26% risk of CI-AKI [12]. Pre-procedure eGFR, creatinine and NGAL levels were not different between RIPC and control groups (48.5 ± 12.0 ml/min vs. 46.6 ± 10.2 ml/min, *p* = 0.391; 1.42 ± 0.58 mg/dl vs. 1.41 ± 0.34 mg/dl, *p* = 0.856; and 136.0 ± 45.0 ng/ml vs. 135.7 ± 43.3 ng/ml, *p* = 0.961, respectively).

**Table 1:** Clinical and angiographic characteristics of the population, remote ischemic preconditioning and control groups.

Variables	Total (n = 102)	RIPC (n = 51)	Control (n = 51)
Age, years	68.9 ± 8.2	67.8 ± 7.6	69.0 ± 8.6
Male, %	48 (47.1)	23 (45.1)	25 (49.0)
**Body mass index, kg/m**^**2**^	25.0 ± 3.4	25.4 ± 3.7	24.6 ± 3.1
<18.5	2 (2.0)	2 (3.9)	0
18.5–24.9	53 (52.0)	25 (49.0)	28 (54.9)
≥25	47 (46.1)	24 (47.1)	23 (45.1)
**Co-morbidities, %**			
Smoker	27 (26.5)	10 (19.6)	17 (33.3)
Hypertension	87 (85.3)	41 (80.4)	46 (90.2)
Dyslipidaemia	49 (48.0)	23 (45.1)	26 (50.1)
Prior MI	7 (6.9)	3 (5.9)	4 (7.8)
Prior CABG	3 (2.9)	2 (3.9)	1 (2.0)
**Ejection Fraction, %**	59.3 ± 11.0	60.2 ± 9.4	58.2 ± 12.5
>60	65 (63.7)	32 (62.7)	33 (64.7)
45–59	22 (21.6)	15 (29.4)	7 (13.7)
30–44	12 (11.8)	4 (7.8)	8 (15.7)
<30	2 (2.0)	0	2 (3.9)
**NYHA Functional class**	2 ± 1	2 ± 1	2 ± 1
I-II	75 (73.5)	36 (70.6)	39 (76.5)
III/IV	26 (25.5)	14 (27.5)	12 (23.5)
**Clinical Diagnosis**			
Insignificant CAD disease	12 (11.8)	8 (15.7)	4 (7.8)
Stable angina	31 (30.4)	15 (29.4)	16 (31.4)
Unstable angina	44 (43.1)	22 (43.1)	22 (43.1)
NSTEMI	15 (14.7)	6 (11.8)	9 (17.6)
**Number of diseased vessels**	2 ± 1	2 ± 1	2 ± 1
0	12 (11.8)	7 (13.7)	5 (9.8)
1	39 (38.2)	19 (37.3)	20 (39.2)
2	21 (20.6)	14 (27.5)	7 (13.7)
3	30 (29.4)	11 (21.6)	19 (37.3)
**Volume of contrast, mls**	196.9 ± 116.	197.5 ± 114.3	196.3 ± 118.8
≤100	19 (18.6)	12 (23.5)	7 (13.7)
101–200	46 (45.1)	23 (45.1)	23 (45.1)
201–300	24 (23.5)	7 (13.7)	17 (33.3)
>300	13 (12.7)	9 (17.6)	4 (7.8)
**Stenting performed, %**	70 (68.6)	35 (68.6)	35 (68.6)
**Mehran's CI-AKI risk score**	11 ± 4	10 ± 3	12 ± 4
Low risk (≤ 5)	1 (1)	1 (2.0)	0
Intermediate risk (6–10)	49 (48.0)	26 (51.0)	23 (45.1)
High risk (11–15)	42 (41.2)	22 (43.1)	20 (39.2)
Very high risk (≥16)	10 (9.8)	2 (3.9)	8 (15.7)
**Vital signs**			
Systolic BP, mmHg	154 ± 26	154 ± 25	155 ± 28
Diastolic BP, mmHg	81 ± 14	80 ± 13	83 ± 16
Heart rate, bpm	75 ± 13	74 ± 12	77 ± 14
**Diabetic medication**			
Insulin	25 (24.5)	12 (23.5)	13 (25.5)
Oral hypoglycaemic agent/diet	77 (75.5)	39 (76.5)	38 (74.5)
**Medications at discharge, %**			
Beta-blockers	33 (32.4)	19 (37.3)	14 (27.5)
ACEi or ARB	57 (55.9)	29 (56.9)	28 (54.9)
Calcium channel blockers	51 (50.0)	23 (45.1)	28 (54.9)
Spironolactone	5 (4.9)	2 (3.9)	3 (5.9)
Aspirin	68 (66.7)	33 (64.7)	35 (68.6)
Clopidogrel	63 (61.8)	33 (64.7)	30 (58.8)
Statin	82 (80.4)	42 (82.4)	40 (78.4)

Values are presented as n (%) and mean ± standard deviation. RIPC = remote ischemic preconditioning, MI = myocardial infarction, CABG = coronary artery bypass grafting, LV = left ventricle, NYHA = New York Heart Association, CAD = coronary artery disease, PCI = Percutaneous coronary intervention, CI-AKI = contrast induced acute kidney injury.

**Table 2:** Baseline laboratory measurements of the cohort, remote ischemic preconditioning and control groups.

Variables	Total (n = 102)	RIPC (n = 51)	Control (n = 51)
eGFR, mls/min/1.73m2	47.6 ± 11.1	48.5 ± 12.0	46.6 ± 10.2
<30	9 (8.8)	4 (7.8)	5 (9.8)
30–59	88 (86.3)	43 (84.3)	45 (88.2)
≥60	5 (4.9)	4 (7.8)	1 (2.0)
Creatinine, mg/dl	1.41 ± 0.48	1.42 ± 0.58	1.41 ± 0.34
BUN, mg/dl	24.6 ± 10.8	24.7 ± 12.8	24.5 ± 8.4
Albumin creatinine ratio	315.7 ± 777.6	345.6 ± 766.4	282.2 ± 800.5
NGAL, ng/ml	135.8 ± 43.9	136.0 ± 45.0	135.7 ± 43.3
cTnT, ng/ml	0.19 ± 0.48	0.22 ± 0.57	0.17 ± 0.37
CKMB, ng/ml	2.89 ± 6.07	2.30 ± 3.59	3.47 ± 7.80
hs-CRP, mg/dl	0.37 ± 0.83	0.32 ± 0.79	0.43 ± 0.88
Hb, g/dl	12.0 ± 2.0	12.1 ± 2.1	11.9 ± 1.9
Hct, %	35.6 ± 6.1	35.5 ± 6.6	35.6 ± 5.6
White blood cell, x10^9^/L	7.4 ± 2.3	7.3 ± 2.3	7.5 ± 2.3
Neutrophil, %	60.6 ± 13.5	60.1 ± 12.1	61.1 ± 14.9
Lymphocyte,%	28.1 ± 10.8	29.3 ± 10.5	27.0 ± 11.0
Monocyte, %	6.7 ± 2.3	6.6 ± 2.2	6.8 ± 2.4
Eosinophil, %	2.7 ± 2.7	2.7 ± 2.7	2.6 ± 2.7
Platelets	241.2 ± 81.9	232.2 ± 74.2	250.1 ± 88.8
HbA1C, %	7.6 ± 1.3	7.8 ± 1.4	7.4 ± 1.2
Total cholesterol, mg/dl	157.0 ± 40.7	155.0 ± 35.4	159.1 ± 45.8
HDL cholesterol, mg/dl	40.8 ± 11.5	40.1 ± 10.0	41.5 ± 13.1
LDL cholesterol, mg/dl	89.9 ± 34.5	86.6 ± 29.6	93.4 ± 39.0
Triglycerides, mg/dl	145.5 ± 91.9	141.8 ± 59.2	149.5 ± 118.3

Values are presented as n (%) and mean ± standard deviation. RIPC = remote ischemic preconditioning, eGFR = estimated glomerular filtration rate by Modification of Diet in Renal Disease formula, cTnT = cardiac troponin T, hs-CRP = high sensitivity C-reactive protein, NGAL = neutrophil gelatinase-associated lipocalin, CKMB = creatinine kinase MB, HBA1c = glycated haemoglobin, Hb = haemoglobin, Hct = haematocrit, BUN = blood urea nitrogen, HDL = high-density lipoprotein, LDL = low-density lipoprotein. To convert the values of serum creatinine to micromoles per litre, multiply by 88.4

The primary outcome CI-AKI occurred in 13.7% (14/102) of patients, 13.7% (7/51) in both RIPC and control groups, with no difference observed (*p* = 1.0). Table 3 describes the study outcomes. There were no statistical differences in the post-procedure creatinine and NGAL levels between RIPC and control groups. There were no significant differences seen in cardiac enzymes and hs-CRP post-procedure between RIPC and control groups. No side effects were expressed by any patient.

**Table 3:** Incidence of Contrast Induced Acute Kidney Injury and changes laboratory measurements.

	Total (n = 102)	RIPC (n = 51)	Control (n = 51)	*p-value
**CI-AKI, %**	14 (13.7)	7 (13.7)	7 (13.7)	1.000
Baseline eGFR <30	2 (2.0)	2 (3.9)	0	1.000
Baseline eGFR 30–59	11 (10.8)	4 (7.8)	7 (13.7)	
Baseline eGFR≥60	1 (1.0)	1 (2.0)	0	
**Serum creatinine, mg/dl**				
Baseline	1.41 ± 0.48	1.42 ± 0.58	1.41 ± 0.34	0.924
at 24 hours	1.43 ± 0.58	1.43 ± 0.73	1.43 ± 0.40	0.924
at 48 hours	1.45 ± 0.57	1.47 ± 0.73	1.43 ± 0.37	0.788
at 72 hours	1.50 ± 0.80	1.55 ± 1.05	1.45 ± 0.43	0.609
**Δ creatinine from baseline, mg/dl**				
to 24 hours	0.02 ± 0.25	0.01 ± 0.28	0.03 ± 0.22	0.684
to 48 hours	0.03 ± 0.32	0.04 ± 0.39	0.02 ± 0.25	0.8
to 72 hours	0.06 ± 0.47	0.10 ± 0.62	0.03 ± 0.24	0.535
**% Δ creatinine from baseline**				
to 24 hours	0.9 ± 16.2	-0.3 ± 16.6	2.1 ± 15.9	0.464
to 48 hours	2.3 ± 18.9	2.0 ± 20.9	2.2 ± 16.9	0.905
to 72 hours	2.8 ± 25.4	3.3 ± 31.3	2.4 ± 18.0	0.88
**Serum NGAL, mg/dl**				
Baseline	135.8 ± 43.9	136.0 ± 45.0	135.6 ± 43.3	0.961
at 6 hours	149.3 ± 48.2	146.6 ± 45.5	152.0 ± 51.1	0.577
at 12 hours	150.4 ± 46.9	148.2 ± 50.0	152.5 ± 44.1	0.648
at 24 hours	147.2 ± 46.7	145.0 ± 50.9	149.5 ± 42.4	0.629
**Δ NGAL from baseline, ng/ml**				
to 6 hours	13.5 ± 30.7	10.6 ± 19.0	16.4 ± 39.1	0.345
to 12 hours	14.5 ± 28.6	12.2 ± 22.3	16.9 ± 33.8	0.405
to 24 hours	11.4 ± 29.7	8.9 ± 25.5	13.9 ± 33.4	0.495
**% Δ NGAL from baseline**				
to 6 hours	12.7 ± 28.1	9.3 ± 14.9	16.1 ± 36.8	0.223
to 12 hours	13.4 ± 26.9	9.8 ± 17.2	16.9 ± 33.8	0.188
to 24 hours	10.9 ± 26.1	7.4 ± 19.1	14.4 ± 31.4	0.177
**cTnT, ng/ml**				
Baseline	0.19 ± 0.48	0.22 ± 0.57	0.17 ± 0.37	0.619
at 6 hours	0.28 ± 0.72	0.31 ± 0.79	0.26 ± 0.66	0.730
at 12 hours	0.44 ± 1.33	0.41 ± 1.02	0.46 ± 1.58	0.863
at 24 hours	0.45 ± 1.24	0.41 ± 0.88	0.49 ± 1.52	0.746
**CKMB, ng/ml**				
Baseline	2.89 ± 6.07	2.30 ± 3.59	3.47 ± 7.80	0.336
at 6 hour	6.21 ± 19.42	4.44 ± 8.76	7.90 ± 25.83	0.376
at 12 hours	8.84 ± 22.33	7.32 ± 13.85	10.30 ± 28.26	0.509
at 24 hours	6.05 ± 13.68	5.03 ± 8.21	7.06 ± 17.56	0.459
**hs-CRP, mg/dl**				
Baseline	0.37 ± 0.83	0.32 ± 0.79	0.43 ± 0.88	0.587
at 24 hours	1.29 ± 1.87	1.20 ± 1.83	1.37 ± 1.91	0.646

Values are presented as n (%) and mean ± standard deviation.

*p-value signifies the difference between RIPC and control groups.

RIPC = remote ischemic preconditioning, cTnT = cardiac troponin T, hs-CRP = high sensitivity C-reactive protein, NGAL = neutrophil gelatinase-associated lipocalin, CKMB = creatinine kinase MB, HBA1c = glycated haemoglobin, Hb = haemoglobin, BUN = blood urea nitrogen, HDL = high-density lipoprotein, LDL = low-density lipoprotein. To convert the values of serum creatinine to micromoles per litre, multiply by 88.4

## Discussion

In this study, RIPC prior to PCI was not effective in preventing CI-AKI in patients with diabetes with pre-existing CKD. The CI-AKI incidence was similar between groups (13.7%), with no significant differences seen in NGAL, cardiac enzymes and hs-CRP.

The protective mechanism of RIPC arises from complex interactions involving various kinase cascades; signal transduction, anti-inflammatory, neuronal and humoral pathways which overall work to reduce the ischemic reperfusion injury to the kidney. However not all studies have shown renoprotective features of RIPC with some showing equivocal results [6, 15, 16]. Er et al demonstrated that RIPC prior to PCI significantly reduced the incidence of CI-AKI in patients with CKD in the subsequent 48 hours [9]. Their CKI-AKI incidence of 40% in the control group was attributed to a high risk population with diabetes and congestive heart failure [9]. The present study which adopted a similar AKI definition to Er et al was performed among patients with diabetes presenting for the diagnosis and treatment of cardiac disease, whom would benefit greatest from RIPC. Although we identified a trend in the CI-AKI incidence between groups, this was not statistically significant, most likely as the study was underpowered due to the lower than expected CI-AKI incidence.

The RIPC technique we adopted differed slightly to Er et al, however was similar to other studies [17, 18]. We used 3 inflation/deflation cycles compared to 4 in Er et al, and did not apply any cuff inflation pressure in the sham preconditioning group. Er et al performed cuff inflation to the patient’s diastolic pressure, followed by a cuff deflation of 10mmHg in the control group. This was unlikely to have resulted in the remarkable difference of the study outcomes. Even when using Kidney Diseases Improving Global Outcomes (KDIGO) [19] AKI definition (defined as an increase in creatinine by ≥0.3 mg/dl within 48 hours or an increase in creatinine to ≥1.5 times baseline), we were unable to show a significant difference in the study outcomes.

The surrogate kidney injury biomarker serum NGAL was measured at baseline up to 24 hours post contrast administration. NGAL is a small 25-kDa protein released from kidney tubular cells after harmful stimuli which allows for early and sensitive detection of CI-AKI [20, 21]. Serum NGAL levels have also been shown to be produced in greater quantities in patients with CKD [22]. We observed an increased baseline NGAL level for all patients of 135.4 ± 43.9 mg/dl; 136.0 ± 45.0 mg/dl in the RIPC group and 135.6 ± 43.3 mg/dl in the control group. Following contrast exposure, NGAL rose at 6 hours and fell by 24 hours, with both groups having the highest percentage change at 12 hours, suggesting the ideal NGAL measurement of between 6 to 12 hours, these results consistent with previous studies [23, 24].

Although the mechanism of CI-AKI in patients receiving contrast exposure is uncertain, there are few postulated mechanisms. Contrast media has direct tubulotoxicity on renal tubular cells giving rise to epithelial vacuolization, cellular membrane damage, cell necrosis and apoptosis and interstitial inflammation [25, 26]. The other mechanism of injury is from contrast-induced vasoconstriction of the vasa recta, which alters the renal haemodynamics and subsequently reducing renal blood flow to the medulla. The outer medulla has high oxygen demands and with the pathophysiological shunting of blood from the medulla to the renal cortex, medullary ischemia-reperfusion injury occurs [27]. Our data studies the occurrence of CI-AKI based on current definitions on the short term changes in creatinine and reciprocally on GFR. Tubular biomarkers such as Vitamin-D-binding protein (VDBP) and Kidney Injury Molecule-1 (KIM-1) describing tubular alterations have been shown to be closely linked to contrast media induced long term clinical consequences, such as major adverse renal events, dialysis need, death and non-elective hospitalization up to 90 days after contrast media exposure [28].

The current best established prophylactic measure for prevention still remains pre and post hydration which increases renal perfusion and counteracts the increased urine volume and osmolar clearance caused by iodinated contrast; their effects made worse by the decrease in renal blood flow as observed in dehydrated states [29, 30].

Although the study was underpowered due to the lower than expected incidence of CI-AKI in our population (13.7% in the overall population and in each group), there are factors to account for this low incidence; including the pre and post intravenous hydration, the use of the least toxic iso-osmolar contrast media, Iodixanol, and the overall stenting rate which was about 69% in each group, higher than the Er et al study, with the improved coronary circulation being an advantage to the patient. The contrast volume was similar between RIPC and control groups (197.5 ± 114.3mls vs. 196.3 ± 118.8mls, p = 0.960) and when divided into contrast volume groups, the control group had 33.3% patients who received 201–300mls of contrast compared to 13.7% in RIPC patients. Despite this, there remained no difference in the CI-AKI incidence between groups. Another difference though unlikely, which may have contributed to our lower CI-AKI incidence was our patient ethnicity (Koreans). However, a Dutch study with similar study methods and design had even lower AKI incidence of 2% in both RIPC and sham conditioning arm [16]. Therefore, although having associative mechanisms, it remains difficult to propose the exact causal relationship between PCI and CI-AKI and subsequently the benefits from RIPC.

### Limitation

This study was performed in a single centre in South Korea and recruited only patients with diabetes with pre-existing chronic kidney disease presenting for PCI. However, this was a randomized, blinded trial with only one independent nurse performing RIPC and being aware of the allocation groups. Secondly, given the small population and the low incidence of CI-AKI compared to what was predicted, the risk of type II error is increased thus influencing the treatment effect, if any, of RIPC. A future larger trial will be required to confirm our findings. Thirdly, this study examined the incidence of CI-AKI and the short term effects following RIPC application and did not look at long-term outcomes. We also were unable to ascertain that the RIPC method used indeed induced ischemia. The postulated pathophysiology of CI-AKI is cytotoxic tubular injury and medullary ischemic injury, although the precise aetiology remains unknown and is far complex. The uncertainty in this aetiology is a potential reason for the failure of RIPC to show renal improvement and protection.

## Conclusions

RIPC applied prior to elective PCI was not effective in preventing CI-AKI in patients with diabetes with pre-existing CKD.

## Supporting Information

S1 File Protocol of Research (Translated to English). This is the study protocol that has been translated from Korean to English.
(DOC)

S2 File IRB-Study Protocol in Korean (DM-NGAL). The is the original study protocol in Korean.
(DOC)

S3 File CONSORT 2010 Checklist. This is the reporting randomized trial checklist for this study.
(DOC)
