# Supplementary material for: Remote Ischemic Preconditioning for the Prevention of Contrast-Induced Acute Kidney Injury in Diabetics Receiving Elective Percutaneous Coronary Intervention
Source: PLoS One. 2016 Oct 10;11(10):e0164256. doi: 10.1371/journal.pone.0164256 (PMC5056748; doi:10.1371/journal.pone.0164256)
Supplement: S1 File — This is the study protocol that has been translated from Korean to English. (DOC) [file pone.0164256.s001.doc]

**Protocol of Research**

**Title: The Effect of Remote Ischemic Preconditioning in diabetics receiving elective PCI**

1. **Backgrounds and Purpose**

Contrast-induced nephropathy (CIN) is a known complication occurring in 2~25% of patients who undergo percutaneous coronary intervention (PCI) and the increment of serum creatinine, of more than 0.3 mg/dl shows worse prognosis. The exact mechanism and pathophysiology of CIN is not well understood. The only proven method to prevent CIN is to hydrate the high risk patients before the PCI. There is still lack of effective medications or intervention to prevent CIN.

Remote Ischemic Preconditioning (RIC) is strategy inducing transient episodes of ischemia by the occlusion of blood flow in non-target tissue such as a limb by manual inflation of the cuff to 200 mmHg for 5 minutes, followed by deflation of 5 minutes to allow reperfusion. This method is non-invasive and inexpensive. The repeated ischemic episodes can confer protection at more remote sites such as the heart, kidney and brain via preventing the injury of endothelial cell or aggregation of platelets and reducing inflammation response. As the exact mechanism is unclear and the systemic effects have not been investigated, RIPC is not applied universally in clinical practice.

Neutrophil gelatinase-associated lipocalin (NGAL) is a biomarker produced in renal tubular cells after acute kidney injury. This is useful to monitor the renal injury.

The objective of this study is to evaluate the efficacy of RIPC in preventing CIN in patients with diabetes with pre-existing chronic kidney disease (CKD) undergoing PCI.

1. **Prior research (references)**

In myocardial infarction, inducing transient remote ischemic episodes (at the forearm) reduces coronary ischemia and thus was beneficial in reducing the infarct area during reperfusion. The protective mechanism arises from the complex interactions involving respective signal transduction, anti-inflammatory, neuronal and humoral pathways differing in response to various ischemic stimuli Therefore, when RIPC is applied before PCI, the kidneys may be protected against ischemia-reperfusion injury and subsequently CIN.

1. **Study subjects and Methods**
2. Diabetic patients (aged between 18 and 85 years old) with nephropathy [estimated glomerular filtration rate (eGFR) ≤60 ml/min/1.73m^2^ or random urine albumin-to-creatinine ratio >300 mg/g] presenting with chest pain and undergoing elective PCI are included. Patients presenting with STEMI are excluded.
3. Hydration with normal saline (intravenous 0.9% NaCl infusion, 60cc/hr) before and after PCI.
4. Patients are not permitted N-Acetylcysteine.
5. Measurement of the baseline sNGAL, creatinine, hs CRP pre- PCI.
6. 1:1 Randomization (Computer-generated block randomization with varying block sizes (6, 4, or 2) used to randomly assign consecutive patients in a 1:1 ratio (single-blind) to treatment: standard PCI (control group), or standard PCI plus RIPC)
7. RIPC is performed by manual inflation of the cuff to 200 mmHg for 5 minutes, followed by deflation of 5 minutes to allow reperfusion and this cycle is performed 3 times. The sham group has the application of the cuff for 30 minutes with no inflation applied.
8. Record the exact amount of contrast (Visipaque)
9. Measure sNGAL at 6hours, 12hours and 24hours after PCI and creatinine at 24hours, 48 hours and 72 hours.
10. Measure CKMB, Troponin T at 6hours, 12hours and 24hours after PCI and hsCRP at 24 hours.


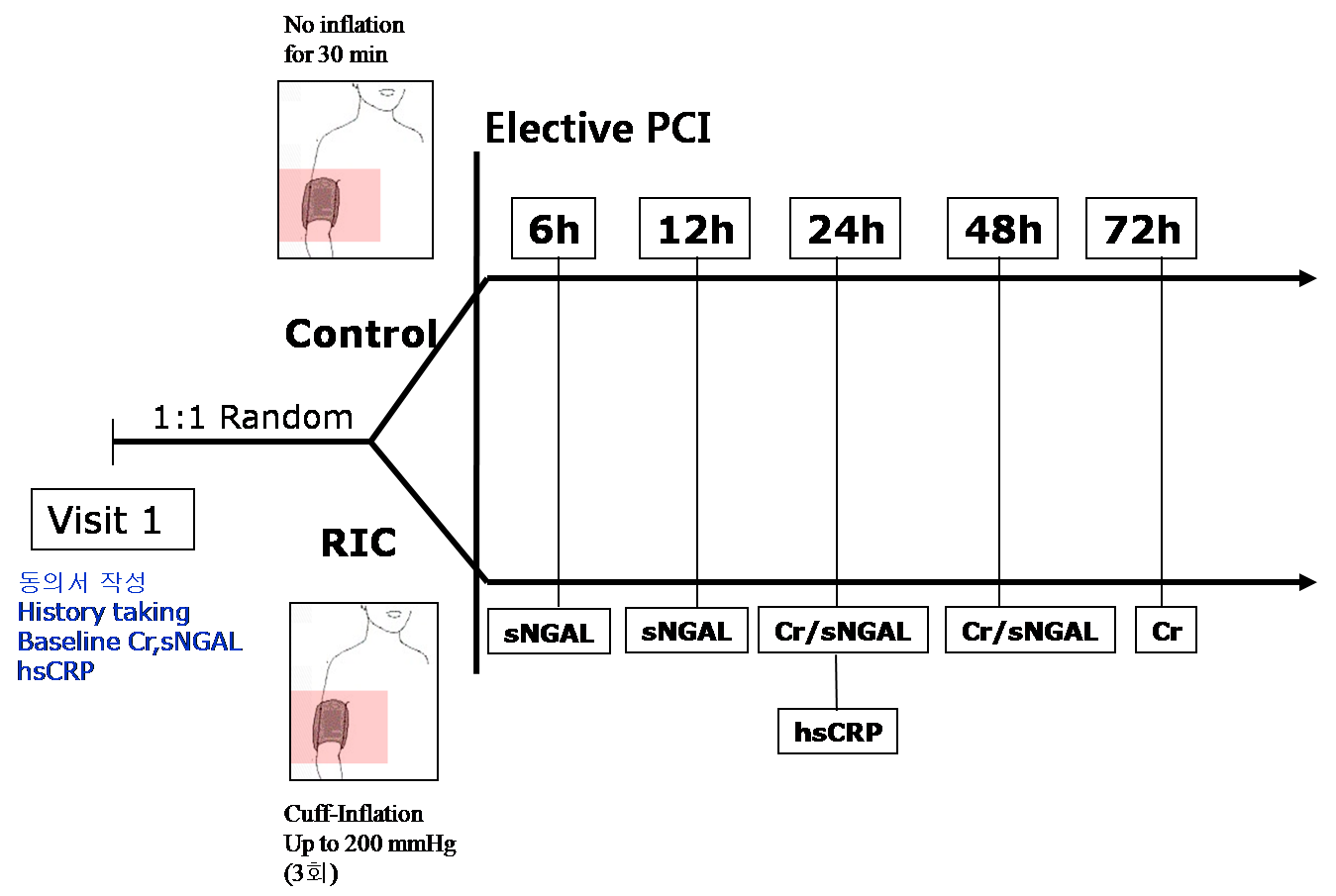


1. **Study end point**

Primary: changes in creatinine within 48 hours after contrast exposure

Secondary: serial changes in creatinine, NGAL, CKMB, Troponin T, hs-CRP over time from baseline

1. **Sample Size**

The sample size was determined on the basis of the primary outcome, post-PCI sNGAL at follow-up. We assumed that remote ischemic conditioning would reduce the sNGAL by 30%; therefore, 100 patients were recruited into the study to enable such a reduction to be detected (α= 0.05; β= 0.2; statistical power = 80%).

1. **Clinical data and blood tests**

Gender, age, height, body weight, history of hypertension, diabetes, chest pain

CBC, serum BUN/creatinine, AS/ALT, HbA1c

Urine albumin/creatinine

sNGAL at 6hours, 12hours and 24hours

creatinine at 24hours, 48 hours and 72 hours

CKMB, Troponin T at 6hours, 12hours and 24hours

hsCRP at 24 hours.

1. **References**

1) Ischemic preconditioning at a distance: reduction of myocardial infarct size by partial reduction of blood supply combined with rapid stimulation of the gastrocnemius muscle in the rabbit., Birnbaum Y, Hale SL, Kloner RA, Circulation. 1997;96(5):1641

2) Transient limb ischemia induces remote ischemic preconditioning in vivo., Kharbanda RK, Mortensen UM, White PA, Kristiansen SB, Schmidt MR, Hoschtitzky JA, Vogel M, Sorensen K, Redington AN, MacAllister R, Circulation. 2002;106(23):2881.

3) Cardioprotective role of remote ischemic periconditioning in primary percutaneous coronary intervention: enhancement by opioid action., Rentoukas I, Giannopoulos G, Kaoukis A, Kossyvakis C, Raisakis K, Driva M, Panagopoulou V, Tsarouchas K, Vavetsi S, Pyrgakis V, Deftereos S., JACC Cardiovasc Interv. 2010 Jan;3(1):49-55.

4) Remote ischaemic conditioning before hospital admission, as a complement to angioplasty, and effect on myocardial salvage in patients with acute myocardial infarction: a randomised trial., Bøtker HE, Kharbanda R, Schmidt MR, Bøttcher M, Kaltoft AK, Terkelsen CJ, Munk K, Andersen NH, Hansen TM, Trautner S, Lassen JF, Christiansen EH, Krusell LR, Kristensen SD, Thuesen L, Nielsen SS, Rehling M, Sørensen HT, Redington AN, Nielsen TT., Lancet. 2010 Feb 27;375(9716):727-34.

5) Does remote ischemic conditioning salvage left ventricular function after successful primary PCI?, Hoole SP, Dutka DP., Expert Rev Cardiovasc Ther. 2011 May;9(5):563-6.

6) Neutrophil Gelatinase–Associated Lipocalin (NGAL) as a Marker of Kidney Damage, Davide Bolignano, Valentina Donato, Giuseppe Coppolino, Susanna Campo, Antoine Buemi, Antonio Lacquaniti, and Michele Buemi, Am J Kidney Dis. 2008 April 3;52:595-60

**7. Study plan**

| **Content** | **schedule** | | | | | | | | | | | | **Cost**  **(1000**  **won)** | **etc** |
| --- | --- | --- | --- | --- | --- | --- | --- | --- | --- | --- | --- | --- | --- | --- |
|  | 1-2 | 3-4 | 5-6 | 7-8 | 9-10 | 11-12 | 1-2 | 3-4 | 5-6 | 7-8 | 9-10 | 11-12 |  |  |
| **IRB** | **.** |  |  |  |  |  |  |  |  |  |  |  | **1,000** |  |
| **Enrollment** |  | **.** | **.** | **.** | **.** | **.** | **.** | **.** | **.** | **.** |  |  | **8,000** |  |
| **RIPC, lab test** |  | **.** | **.** | **.** | **.** | **.** | **.** | **.** | **.** | **.** |  |  | **5,000** |  |
| **Data collection** |  |  | **.** | **.** | **.** | **.** | **.** | **.** | **.** | **.** | **.** | **.** | **4,000** |  |
| **Statistical analysis, writing, submission** |  |  |  |  |  |  |  |  |  |  | **.** | **.** | **2,000** |  |
| **Progress (%)** | **50** | | | **70** | | | **80** | | | **100** | | |  |  |
| **Cost (1000 won)** | **8,000** | | | **5,000** | | | **5,000** | | | **2,000** | | |  |  |
