# Supplementary material for: Remote Ischemic Preconditioning for the Prevention of Contrast-Induced Acute Kidney Injury in Diabetics Receiving Elective Percutaneous Coronary Intervention
Source: PLoS One. 2016 Oct 10;11(10):e0164256. doi: 10.1371/journal.pone.0164256 (PMC5056748; doi:10.1371/journal.pone.0164256)
Supplement: S2 File — The is the original study protocol in Korean. (DOC) [file pone.0164256.s002.doc]

**연 구 내 용**

**연구제목:** The Effect of Remote Ischemic Preconditioning in diabetics receiving elective PCI

**1. 연구배경 및 목적**

조영제로 인한 급성신장병(contrast-induced nephropathy, CIN)은 경피적 관상동맥 중재시술(percutaneous coronary intervention, PCI)후 2 ~ 25% 발생하는 것으로 알려져 있으며. 관상동맥 촬영 후에 혈청 creatinine이 0.3 mg/dl 만 상승해도 예후가 나쁜 것으로 알려져 있다. CIN의 병태생리는 혈관과 tubule 에서의 복잡한 상호작용에 의해 일어나는 것으로 이해되고 있으며 현재까지 정확한 기전은 모르는 것으로 알려져 있다. 때문에 CIN 의 예방은 당뇨병성 신장병(diabetic nephropathy)같은 고위험군을 찾아서 시술 전에 정맥으로 충분한 수액을 공급해 주는 정도이며 그 외에 아직까지 뚜렷한 효과가 있는 약물이나 중재방법은 알려져 있지 않다.

원격허혈조절(Remote Ischemic Conditioning, RIC)은 팔이나 다리에 혈압을 재는 Cuff를 감고 200 mmHg까지 올려서 원위부에 허혈상태를 만든 후 5분간 그대로 두었다가 풀어서 다시 혈류를 회복시키는 방법이다. 이는 비침습적이고 추가 비용이 들지 않는 간단한 방법으로 심장과 신장, 뇌 등의 허혈로 인한 손상을 효과적으로 줄여주는 것으로 보고되고 있다. 원격 허혈 조절의 기전은 혈관 내피세포의 손상을 막고 혈관 염증반응을 감소시키며 혈소판 응집을 억제 하여 일어난다고 보고 있으나 아직까지 그 기전은 불명확하고, 경색부위 자체 이외의 다른 전신에 대한 효과는 연구된 바 없으며, 현재까지 임상에서 일반적으로 적용되고 있지는 않다.

Neutrophil gelatinase-associated lipocalin(NGAL)은 급성 신손상시에 신장의 tubular cell 에서 만들어지는 biomarker 로 알려져 있으며 creatinine 보다 조기에 신장의 손상 정도를 알 수 있다.

이 연구는 당뇨병성 신장병이 있는 관상동맥 질환환자에서 계획된 PCI후 발생할 수 있는 CIN을 RIC가 얼마나 예방해 줄 수 있는지를 NGAL 이라는 biomarker 를 이용하여 알아보고자 하였다.

**2. 연구의 배경이 되는 선행 연구결과**

여러 연구에서 심근경색에서의 재관류 시술시 관상동맥에 인위적으로 일시적인 관상동맥 허혈을 유발 후 재관류를 시행했을 때 심근경색 허혈손상 부위를 줄이는 긍정적인 효과를 보였으며, 심장에서 원위부인 전완 등에 허혈을 유발한 연구에서도 비슷한 결과를 보여주고 있습니다.

이에 대한 기전은 명확하게 밝혀지지 않았지만, 근육 허혈을 일으킴으로 해서 분비되는 여러가지 호르몬 매개체와 신경학적 기전으로 혈관 내피를 보호하고, 혈관의 염증발생을 저하시키며, 혈소판의 응집을 억제하는 것 들이 주 기전으로 여겨지고 있습니다. 이 연구는 pilot study 로서 신장의 신혈관에도 동일한 기전의 혈관 보호효과가 적용되어 CIN을 줄일 수 있을 것으로 기대합니다.

**3. 연구 대상 및 방법**

1) Elective PCI 가 예정되어 있는 환자 중에서 당뇨병성 신장병(=albumin/creatinine > 300 or eGFR ≤ 60)이 있는 사람을 대상으로 한다(18세 이상, 85세 이하). STEMI 는 제외한다.

2) PCI 전후로 6시간 normal saline으로 hydration (60cc/h) 한다.

3) N-Acetylcysteine 은 복용하지 않는다.

3) PCI 당일 baseline sNGAL, creatinine, hsCRP를 측정한다.

4) 1:1 randomization 한다 (Computer-generated block randomisation with varying block sizes (6, 4, or 2) is used to randomly assign consecutive patients in a 1:1 ratio (single-blind) to treatment: standard PCI (control group), or standard PCI plus RIC)

5) RIC group은 PCI 1시간 전까지 RIC를 3cycle 시행한다 (BP cuff를 200 mmHg까지 올려서 5분 지속시킨 후 풀고 5분 휴식하는 것을 1 cycle 로 해서 3회 반복한다). Control group 은 BP cuff 를 상박에 위치시킨 압력을 가하지 않고 30분간 유지한다.

6) Visipaque 를 이용하여 PCI를 시행한다. 사용한 양을 정확히 기록한다.

7) PCI 6h, 12h, 24h 후에 sNGAL을 측정하고 creatinine은 24h, 48h, 72h 후에 측정한다.

8) PCI 후 CKMB, Troponin T를 6h, 12h, 24h 에 측정한다. 24h 에 hsCRP 같이 측정한다.


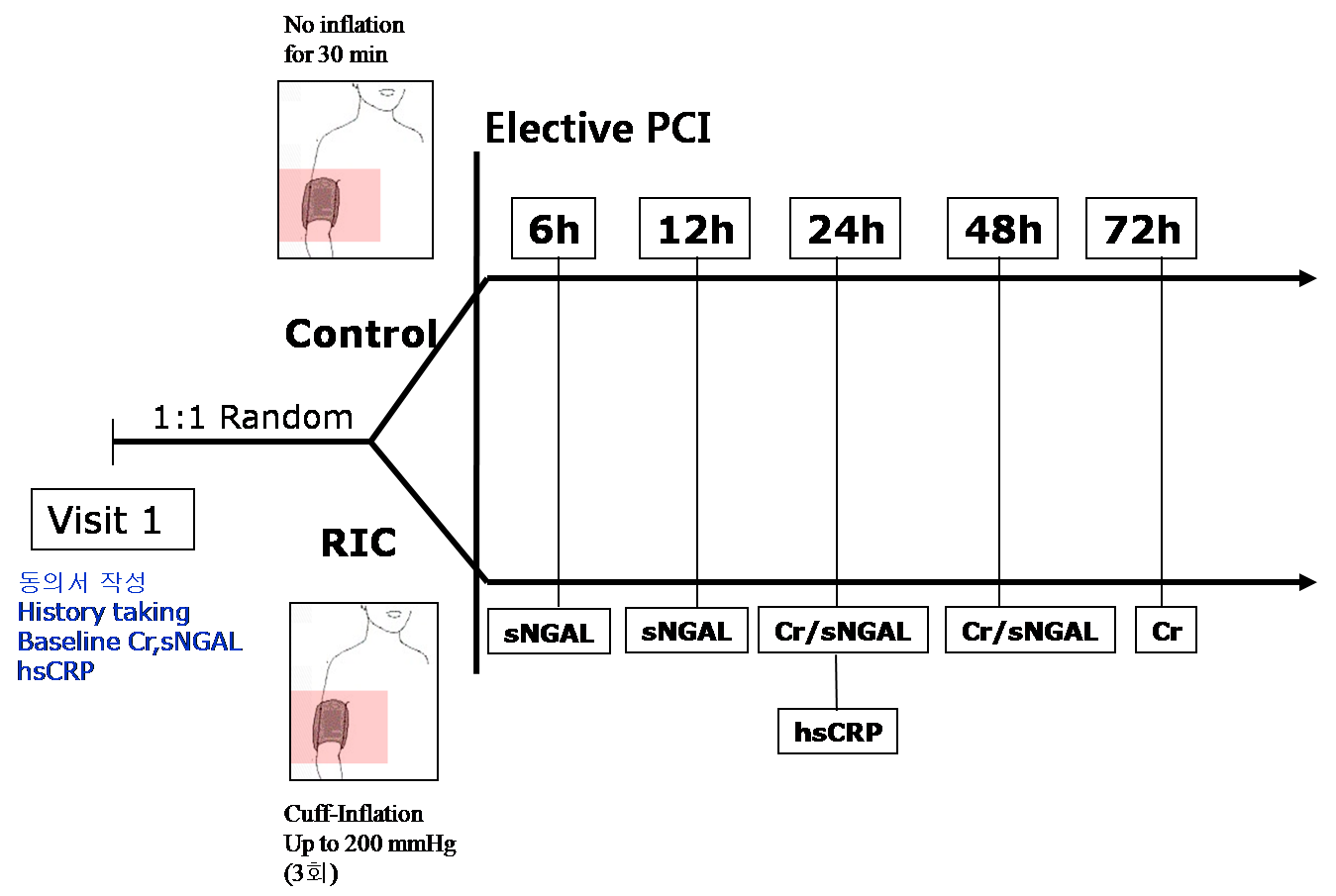


**4. Study end point**

Primary: PCI 후 sNGAL, crearinine 의 변화

Secondary: CKMB, Troponin T, hs-CRP 의 변화

**5. Sample Size**

The sample size was determined on the basis of the primary outcome, post-PCI sNGAL at follow-up. We assumed that remote ischemic conditioning would reduce the sNGAL by 30%; therefore, 100 patients were recruited into the study to enable such a reduction to be detected (α= 0.05; β= 0.2; statistical power = 80%).

**6. 임상정보 수집 및 기타혈액검사**

성별, 나이, 키, 몸무게, 고혈압 병력, 당뇨 병력, 운동시와 휴식시 협심증 증상 여부

CBC, 혈청 BUN/Creatinine, AST/ALT, HbA1c

소변 albumin/creatinine

혈청 NGAL을 시술 6, 12, 24시간 후 측정

Creatinine 를 24, 48, 72시간 후 측정

혈청 CKMB, Troponin T 를 시술 6, 12, 24시간 후 측정

hs-CRP를 24시간 후 측정

**7. 참 고 문 헌**

1) Ischemic preconditioning at a distance: reduction of myocardial infarct size by partial reduction of blood supply combined with rapid stimulation of the gastrocnemius muscle in the rabbit., Birnbaum Y, Hale SL, Kloner RA, Circulation. 1997;96(5):1641

2) Transient limb ischemia induces remote ischemic preconditioning in vivo., Kharbanda RK, Mortensen UM, White PA, Kristiansen SB, Schmidt MR, Hoschtitzky JA, Vogel M, Sorensen K, Redington AN, MacAllister R, Circulation. 2002;106(23):2881.

3) Cardioprotective role of remote ischemic periconditioning in primary percutaneous coronary intervention: enhancement by opioid action., Rentoukas I, Giannopoulos G, Kaoukis A, Kossyvakis C, Raisakis K, Driva M, Panagopoulou V, Tsarouchas K, Vavetsi S, Pyrgakis V, Deftereos S., JACC Cardiovasc Interv. 2010 Jan;3(1):49-55.

4) Remote ischaemic conditioning before hospital admission, as a complement to angioplasty, and effect on myocardial salvage in patients with acute myocardial infarction: a randomised trial., Bøtker HE, Kharbanda R, Schmidt MR, Bøttcher M, Kaltoft AK, Terkelsen CJ, Munk K, Andersen NH, Hansen TM, Trautner S, Lassen JF, Christiansen EH, Krusell LR, Kristensen SD, Thuesen L, Nielsen SS, Rehling M, Sørensen HT, Redington AN, Nielsen TT., Lancet. 2010 Feb 27;375(9716):727-34.

5) Does remote ischemic conditioning salvage left ventricular function after successful primary PCI?, Hoole SP, Dutka DP., Expert Rev Cardiovasc Ther. 2011 May;9(5):563-6.

6) Neutrophil Gelatinase–Associated Lipocalin (NGAL) as a Marker of Kidney Damage, Davide Bolignano, Valentina Donato, Giuseppe Coppolino, Susanna Campo, Antoine Buemi, Antonio Lacquaniti, and Michele Buemi, Am J Kidney Dis. 2008 April 3;52:595-60

**8. 연구추진일정표**

| **연 구 내 용** | **연 구**  **책임자** | **추 진 일 정** | | | | | | | | | | | | **연구비**  **(천원)** | **비고** |
| --- | --- | --- | --- | --- | --- | --- | --- | --- | --- | --- | --- | --- | --- | --- | --- |
|  |  | 1-2 | 3-4 | 5-6 | 7-8 | 9-10 | 11-12 | 1-2 | 3-4 | 5-6 | 7-8 | 9-10 | 11-12 |  |  |
| **IRB 심의** | **신은석** | **.** |  |  |  |  |  |  |  |  |  |  |  | **1,000** |  |
| **환자등록** | **상동** |  | **.** | **.** | **.** | **.** | **.** | **.** | **.** | **.** | **.** |  |  | **8,000** |  |
| **원격 허혈 조절 및 혈액/소변검사** | **상동** |  | **.** | **.** | **.** | **.** | **.** | **.** | **.** | **.** | **.** |  |  | **5,000** |  |
| **임상자료수집 및 기록** | **상동** |  |  | **.** | **.** | **.** | **.** | **.** | **.** | **.** | **.** | **.** | **.** | **4,000** |  |
| **통계분석, 결과도출 및 발표** | **상동** |  |  |  |  |  |  |  |  |  |  | **.** | **.** | **2,000** |  |
| **사업진도(%)** |  | **50** | | | **70** | | | **80** | | | **100** | | |  |  |
| **연구비(천원)** |  | **8,000** | | | **5,000** | | | **5,000** | | | **2,000** | | |  |  |

**9. SCHECULE OF MEASUREMENTS**

| Measurement Component | Baseline | Post-PCI | | | | |
| --- | --- | --- | --- | --- | --- | --- |
|  |  | 6h | 12h | 24h | 48h | 72h |
| Informed consent | X |  |  |  |  |  |
| Inclusion/Exclusion criteria | X |  |  |  |  |  |
| Clinical/Medical history | X |  |  |  |  |  |
| Vital Status & Physical exam | X |  |  |  |  |  |
| Weight & Height | X |  |  |  |  |  |
| ECG(12 lead) | X | X |  | X |  |  |
| CKMB, Troponin T | X | X | X | X |  |  |
| CBC, electrolyte | X |  |  |  |  |  |
| BUN/Cr, | X |  |  | X | X | X |
| Total cholesterol, triglyceride, HDL, LDL | X |  |  |  |  |  |
| sNGAL | X | X | X | X |  |  |
| hs-CRP | X |  |  | X |  |  |
| HbA1C | X |  |  |  |  |  |
| Current Medication | X |  |  |  |  |  |
| CV event | X | X | X | X | X | X |

**10. 소요 예산**

(단위 : 천원)

| **비목**  **번호** | **비 목 구 분** | | **금 액** | **비 율(%)** | **비 고** |
| --- | --- | --- | --- | --- | --- |
| **1** | **인건비** | | **7,800** | **39** | ***총 연구비의 60% 이내** |
| **2** | **연구**  **활동비** | **회의비** | **0** | **0** | ***총 연구비의 5% 이내** |
|  |  | **논문 게재료** | **200** | **1** |  |
|  |  | **유인물비** | **0** | **0** |  |
|  |  | **재료 구입비** | **0** | **0** |  |
| **3** | **직접성**  **경비** | **장비 사용료**  **(임차료)** | **0** | **0** |  |
|  |  | **검사료** | **10,000** | **50** | **혈액검사 비용** |
|  |  | **연구 기기비** | **0** | **0** |  |
| **4** | **간접비** | | **2,000** | **10** |  |
|  | **연구비 총액** | | **20,000** | **100** |  |
